# Attention-Deficit/Hyperactivity Disorder and Parenting: Toward a Cognitive/Schema Model

**DOI:** 10.1192/j.eurpsy.2022.1073

**Published:** 2022-09-01

**Authors:** M. Miklósi, V. Nagy, S. Oláh

**Affiliations:** 1 Eötvös Loránd University, Department Of Developmental And Clinical Child Psychology, Budapest, Hungary; 2 Heim Pál Children’s Hospital, Mental Health Centre, Budapest, Hungary

**Keywords:** adhd, attention-deficit/hyperactivity disorder, parenting, schema modes

## Abstract

**Introduction:**

Attention-Deficit/Hyperactivity Disorder (ADHD) runs in families; however, there are mixed results on the interaction effects of parent’s and child’s psychopathology on parenting qualities. Cognitive/schema therapy is a promising treatment approach for adult ADHD; we know little about the effect of cognitive factors on parenting, however.

**Objectives:**

We aimed to fill this gap by exploring the role of dysfunctional schema modes in the associations between adult ADHD symptoms, child’s externalizing symptoms, and perceived parental competence in a dimensional approach.

**Methods:**

A community sample of parents (N=100, mean age=38.25 years, SD=5.73) filled out online questionnaires assessing ADHD symptoms (Adult ADHD Self-Report Scale), dysfunctional schema modes (Schema Mode Inventory), perceived parental competence (Parental Sense of Competence Scale), and child’s psychopathology (Strength and Difficulties Questionnaire).

**Results:**

In a multivariate model, higher levels of parental ADHD symptoms were related to higher levels of dysfunctional schema modes. However, neither the child’s externalizing symptoms nor the interaction term of parent’s symptoms by the child’s symptoms had any effect on dysfunctional schema modes. Furthermore, the child’s externalizing symptoms had a direct negative association with parental self-efficacy beliefs, whereas the relationship between adult ADHD symptoms and parental self-efficacy was mediated by the detached and overcompensating dysfunctional schema modes.

**Conclusions:**

Our results suggest that the activation of dysfunctional schema modes is related to the parent’s but not the child’s psychopathology. The activation of dysfunctional schema modes may play an important role in the self-efficacy beliefs of parents with ADHD. Targeting that cognitive factors may enhance the effect of behavioral parent training programs.

**Disclosure:**

This research was supported by National Research, Development, and Innovation Office (NKFIH) OTKA-PD-134849 grant.

